# Elasto-Static Analysis of Composite Restorations in a Molar Tooth: A Meshless Approach

**DOI:** 10.3390/polym16040458

**Published:** 2024-02-07

**Authors:** Farid Mehri Sofiani, Behzad V. Farahani, Jorge Belinha

**Affiliations:** 1 FEUP, Faculty of Engineering, University of Porto, Dr. Roberto Frias Street, 4200-465 Porto, Portugal; 2 ISEP, Instituto Superior de Engenharia do Porto, Mechanical Engineering Department, School of Engineering, Polytechnic Porto, Dr. António Bernardino de Almeida Street, 431, 4249-015 Porto, Portugal; job@isep.ipp.pt

**Keywords:** numerical modelling, meshless methods, bruxism, dental composites, stress analysis, FEM

## Abstract

Dental caries and dental restorations possess a long history and over the years, many materials and methods have been invented. In recent decades, modern techniques and materials have brought complexity to this issue, which has created the necessity to investigate more and more to achieve durability, consistency, proper mechanical properties, efficiency, beauty, good colour, and reduced costs and time. Combined with the recent advances in the medical field, mechanical engineering plays a significant role in this topic. This work aims at studying the elasto-static response of a human molar tooth as a case study, respecting the integral property of the tooth and different composite materials of the dental restoration. The structural integrity of the case study will be assessed through advanced numerical modelling resorting to meshless methods within the stress analysis on the molar tooth under different loading conditions. In this regard, bruxism is considered as being one of the most important cases that cause damage and fracture in a human tooth. The obtained meshless methods results are compared to the finite element method (FEM) solution. The advantages and disadvantages of the analysed materials are identified, which could be used by the producers of the studied materials to improve their quality. On the other hand, a computational framework, as the one presented here, would assist the clinical practice and treatment decision (in accordance with each patient’s characteristics).

## 1. Introduction

Oral health is integral to human well-being, contributing significantly to overall quality of life. Maintaining healthy teeth enhances sensory perception, which plays a crucial role in overall health and well-being. However, oral diseases, which range widely, cause pain and disorders for many people each year. Dental erosion, tooth decay, periodontal (gum) disease, and mechanical damages are the most common dental disorders.

Dental restoration as a concept can be first found recorded 1400 years ago in China, mainly motivated by dental caries. For modern humans, due to the changes in lifestyle and diet, dental carries have become more prevalent than in the past, which motivated scientists to find and develop new materials and techniques to solve this problem.

Composites are extensively used in modern dentistry. The major reasons can be related to their aesthetic appeal and strong bonding capabilities. To ensure optimal outcomes in clinical applications, these materials must possess appropriate physico-chemical properties. Among all factors, the type of polymeric matrix, filler–matrix composition, and their respective percentages play a vital role in influencing mechanical behaviour [1].

One of the threats to a normal tooth is fracture due to several situations, such as self-contact between teeth or abrupt impact with an external object. One of the most important cases that induces damage and fracture in a human tooth is bruxism [2]. It is considered as a mechanical risk factor for dental damage that has its root cause in the central nervous system (CNS), which forces the jaw muscle to move in a repetitive way, leading to the grinding and clenching of the teeth. Bruxism becomes an important issue due to its capability to cause fracture or tooth wear. It can happen either during the awake period or during the sleep period. It is often related to stress and anxiety, and it can also be caused by misalignment of the teeth or jaw, or by certain medications affecting the CNS. This common phenomenon is still under investigation, and there are many unknown reasons for bruxism. However, this paper intends to present a numerical stress analysis on a molar tooth in which the boundary conditions are prescribed conforming to the bruxism phenomenon; it highlights the structural integrity assessment of the tooth in the presence of the bruxism loading condition and predicts the potential damaged areas.

The development of computational tools requires comprehensive knowledge of the material properties of the human oral system, particularly the jawbone (mandible). Nonetheless, determining accurate biomechanical properties for living tissues, especially bone tissue in numerical models, remains a challenging endeavour, c.f. [3,4,5,6].

Biomechanical simulation of actual dental restorations can anticipate the mechanical behaviour of treated teeth, contributing to optimal clinical solutions for patients. To conduct biomechanical analyses of tooth restorations, it is imperative to establish a numerical model that accurately represents the biomechanical structure. Considering the complexity of the structural system including the tooth–bone assembly, constructing the numerical model necessitates a degree of simplification. Therefore, the literature [7] reveals numerous numerical models incorporating reasonable simplifications, including approximations of geometric shapes, material properties, displacement constraints, and mechanical load systems.

Computational methods have grown even more challenging to find the most accurate solutions to these problems. Amongst all, the finite element method (FEM) is well known for its versatility. It has the potential to address the biomedical models that are challenging due to structural complexity [8]. Finite element analysis (FEA) facilitates the execution of repeatable experiments without the need for ethical approval, and design studies can be easily modified to meet specific requirements. However, there are inherent limitations as FEA represents a computerised approximation of in vitro studies. Stress analysis typically occurs under static loading scenarios, and the mechanical properties of teeth are often simplified as isotropic, homogeneous, and linearly elastic, deviating from in vivo conditions. Moreover, geometric oversimplification may impact result accuracy.

In this regard, Romanyk et al. [9] performed a stress analysis on a molar tooth through the FEM to assess how the load application and mesh density convergence measures could affect the results for a representative crown geometry. Jorge et al. [10] used the FEM formulation to simulate various force lines in action that would influence the distribution and intensity of orthopaedic and orthodontic forces in the maxilla. The obtained results were reported in displacement and von Mises stress variations. Ritthiti et al. [11] performed a 3D FEA to study the effect of stress on class I bulk-fill composite restoration due to the occlusal cyclic load. Singhal et al. [12] performed a clinical analysis to evaluate the cuspal deflection caused by dissimilar composite materials if various insertion techniques are used.

People with bruxism can develop wedge-shaped tooth defects. The forceful grinding or clenching of teeth in bruxism creates excessive pressure on the teeth and their supporting structures, which may contribute to the formation of wedge-shaped defects in combination with other factors. In this regard, Sakhabutdinova et al. [13] performed a numerical analysis to assess the stress state on a tooth model in the presence of two wedge-shaped defects and different restorative materials. Furthermore, Brailko et al. [14] numerically studied the influence of size and location of wedge-shaped defects of teeth on stress and strain state of restorative material on the basis of biomechanical analysis. Moreover, V-shaped tooth defects filled with universal nanohybrid composite using incremental technique were studied by FEM modelling [15]. The study inferred that universal nanohybrid composites would be better choice for filling of V-shaped tooth defects due to the lower microleakage.

On the other hand, meshless methods are proficient in studying complex structural models. The high-order continuity of the built-shape functions contributes to accomplishing smoother internal variables such as strain/stress fields. It can be proficiently employed to resolve large deformation problems. It allows for the insertion of more nodes locally where discretisation refinement is obligatory, with no extra computational cost [16].

In both FEM and meshless methods, the general frame of the solution procedure is the same and the nodal density of the discretisation, as well as the nodal distribution (to be either regular or irregular), can affect the accuracy of the performance. Increasing the number of nodes can, usually, lead to a more accurate result [16].

One of the main factors affecting the acceptance of a numerical technique is computational cost. Commonly, the cost is measured for a prescribed accuracy for a problem. Therefore, by considering that more nodes would increase the computational cost, it is necessary to optimise the cost meticulously without a negative effect on accuracy. Furthermore, it is usually recommended to set a high nodal density close to the locations in which there are expected stress concentrations, such as crack tips, convex, natural, and essential boundaries, domain discontinuities, etc. [16].

In the last few decades, several interpolator meshless methods were implemented, such as the point interpolation method (PIM) [17], the radial point interpolation method (RPIM) [18], the natural neighbour finite element method (NNFEM) [19], the meshless finite element method (MFEM) [20], and more recently the natural neighbour radial point interpolation method (NNRPIM) and the radial natural element method (NREM) [16]. Meshless methods have been used in different engineering areas in civil [21], biomechanical [22,23], and mechanical engineering [24].

Defining material properties, functional loading, applied boundary conditions, and geometrical details are important factors influencing numerical results accuracy. This work highlights the significance of accurately assigning material properties to teeth as they form the basis for stress measurements. Therefore, this study aims at investigating different composite dental materials’ behaviour under certain load cases (occlusal and bruxism) in a condition that one side of the molar tooth is free, and, on the opposite side, there is a tooth, just next to the molar tooth being analysed.

In this work, the RPIM and NNRPIM formulations are employed to analyse the elasto-static response of a molar tooth under bruxism loading conditions. In the studied model, the cortical and trabecular bone surrounds a normal molar tooth in the root, and the tooth itself is constituted of heterogeneous material, such as enamel, dentine, pulp, and periodontal ligament. In the structural model, a part of a tooth is replaced by some dental restorative composites. Therefore, a stress analysis is monitored on different parts of the model. In addition, the model is solved through the FEM formulations to numerically validate the meshless method solutions.

The outlined methodology and findings from the implementation of meshless methods in this study tend to fill the gaps in modern dental science. These advancements carry substantial clinical implications, providing dentists with insights into the physical details of bruxism. The detailed points explained in this research are especially beneficial for clinicians/students/interns who may not possess enough experience with numerical modelling. This work is uniquely positioned to facilitate their understanding of problem definitions and the inherent constraints associated with study results. Particularly, the examination of material responses under distinct loading conditions would provide a precise measurement of the material’s stress response.

## 2. Meshless Methods

In this study, the radial point interpolation meshless method (RPIM) and its natural neighbour extension (NNRPIM) formulations have been employed to analyse the elasto-static response of the case studies defined in the previous section. Therefore, a 2D plane strain deformation theory was considered as extensively described in the previously published papers [16].

This work conforms to the mathematical basis and integration schemes described in the previous works conducted by the authors [16]. A computational algorithm based on the radial point interpolation (RPI) formulations for the elasto-static analysis of the studied model has been implemented in MATLAB^©^ by the authors. It must be noted this study follows mathematical formulations documented in the previously published work [16].

## 3. Material and Model Specification

**Figure 1:**
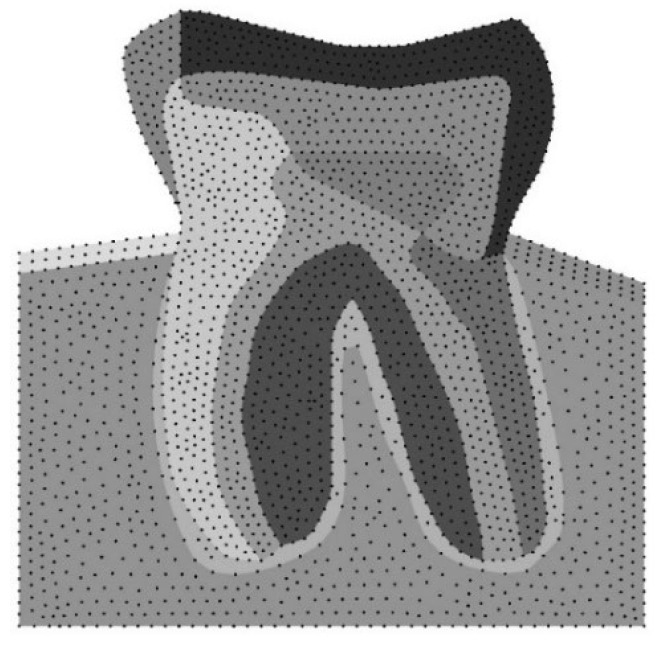
A 2D numerical model of a human molar tooth, meshless method representation.

In this study, a 2D model of a human molar tooth has been introduced for detailed investigation, as illustrated in Figure 1. It is important to note that the model’s geometry has been generated from an X-ray captured in a real clinical case. Consequently, it is feasible to conduct a 2D numerical analysis. Under certain loading situations, load cases simulating normal occlusal loads and paranormal loads, such as bruxism, are analysed. Table 1 reports the mechanical properties of the human tooth. Notice that numerous factors influence the measurement of mechanical properties in human bony structures, including teeth. Among these factors, the structure and scale of human bones are particularly significant. Regarding enamel material, it must be mentioned that it is a natural substance that is biocompatible with the body. It is considered the hardest material serving the outer layer of the dental crown. It is highly mineralised and designed to withstand the forces of chewing and biting. It is durable, possessing sufficient resistance against wear. It provides a strong protective covering for teeth.

**Figure 2:**
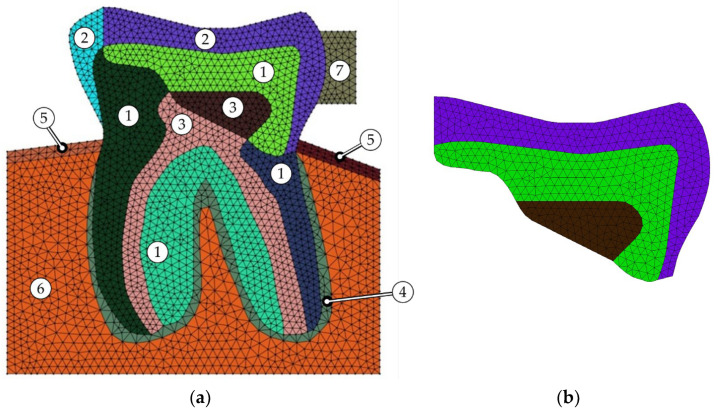
The 2D numerical model respecting the FE mesh representation; (**a**) molar tooth material identification and (**b**) three sections in which composite restorations are filled with.

The material model is heterogeneous, constituted of several sections to feasibly manage material properties and mesh homogeneously. As illustrated in Figure 2a, the model is introduced into the mandibular bone (both cortical and trabecular tissues are represented). On the left side of the studied molar tooth, there is a void representing an absence of a tooth. This case signifies a real clinical case, in which the neighbouring tooth was removed to insert an implant. Then, during the night, due to the absence of the neighbouring tooth and due to bruxism (the patients typically present evident signs of bruxism), the central tooth (studied tooth, the one represented in the figure) would be fractured. On the right side, there is a neighbouring normal molar tooth characterised by fictitious material, referring to the one identified as #7 in Figure 2a. The crown of this tooth is considered in this analysis in which it is covered with enamel, hence, its material property can be defined the same as enamel, referring to Table 1. Nevertheless, all sections are represented by a material as identified in Figure 2a and stated in Table 1.

As shown in Figure 2b, when the restored tooth is being analysed, sections 1, 2, and 3 are assigned three commercial restorative dental composites, the 3M™ Z100™, 3M™ Filtek™ Z250^TM^, and Kerr Herculite XRV Ultra^TM^, with the material properties reported in Table 1. It must be noted that the Poisson’s ratio of the studied materials has been considered according to the literature, c.f. [25]. Moreover, it has been inferred that the flexural or bending modulus of elasticity is equivalent to the tensile modulus (Young’s modulus) or compressive modulus of elasticity for very small strains in isotropic materials—like glass, metal, or polymer [26]. The rest of the material properties have been used as reported in [27], originating from the manufacturers 3M^©^ (3M Center, Saint Paul, MN, USA) and Kerr^©^ (KERR Manufacturing Company, Orange, CA, USA).

**Table 1:** Mechanical properties attributed to the materials of a human tooth and the composites used in this study.

Human Tooth									
Identification	Material		E (GPa)	ν		$\sigma_{t}$ (MPa)		$\sigma_{c}$ (MPa)	
1	Dentine		18.60 [28]	0.31 [28]		Avg.: 24 [29]		Avg.: 310 [29]	
2	Enamel		41.00 [28]	0.30 [28]		Avg.: 108 [29]		Avg.: 279 [29]	
3	Pulp		0.003 [28]	0.45 [28]		-		-	
4	Periodontal ligament		0.07 [30]	0.45 [30]		-		-	
5	Cortical bone		13.70 [30]	0.30 [30]		min: 133 [29]		-	
6	Cancellous bone		1.37 [30]	0.30 [30]		min: 75 [29]		-	
7	Fictitious material		41.00	0.30		-		-	
Composite restorations									
Material	Manufacturer	Type	Composition		Filler	$E\;\left(\mathrm{GPa}\right)$	ν	$\sigma_{t}\left(\mathrm{MPa}\right)$	$\sigma_{c}\left(\mathrm{MPa}\right)$
3M™ Filtek™ Z250^TM^	3M ESPE	Microhybrid	Matrix: BisGMA, UDMA, TEGDMA Filler: Zerconia, silica		60%	11.0	0.31	85	405
3M™ Z100™	3M ESPE	Microhybrid	Matrix: BisGMA, TEGDMA Filler: Zerconia, silica		66%	14.5	0.30	105	470
Herculite XRV Ultra^TM^	Kerr	Nanohybrid	Matrix: BisGMA, TEGDMA Filler: PPF, barium glass, silica nanofiller		59%	8.2	0.30	137	349

## 4. Numerical Modelling

This elasto-static analysis aims to understand the stress field of the studied tooth due to normal occlusal and bruxism loads. Therefore, regarding the natural boundary conditions, four load cases are imposed representing occlusal and bruxism load cases:

- Occlusal right vertical (ORV);
- Occlusal left vertical (OLV);
- Bruxism right vertical (BRV);
- Bruxism left vertical (BLV).

**Figure 3:**
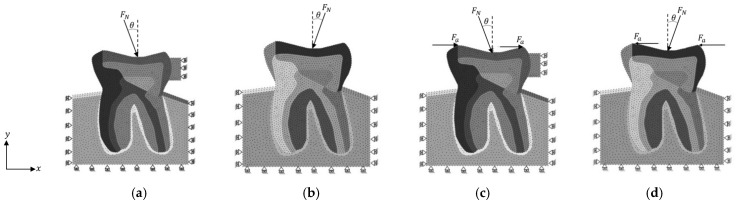
Boundary condition applications of different case studies, (**a**) ORV, (**b**) OLV, (**c**) BRV, and (**d**) BLV.

For the essential boundary conditions, the lower edge is fixed in the *y*-direction, right and left edge of the trabecular bone are fixed in the *x*-direction, c.f. Figure 3.

Nevertheless, in two load cases—BRV and ORV—in which the forces are oriented from left to right, the fictitious material becomes involved in the problem and assumes the material properties of enamel and its boundary condition are fixed in the *x*-direction. However, in load cases BLV and OLV, there is no fictitious material, see Figure 3.

It is important to mention that each force is divided by 5 and applied into five nodes, which, overall, provides the total force being assumed to impose. Carey et al. [31] determined a force range of F=25 to 450 N as an occlusal load to investigate the relationship between applied occlusal load and articulating paper mark area. Furthermore, Vasudeva and Bogra [32] used a force range of F=100 to 500 N in their FEM study on the effect of occlusal restoration and loading on the development of abfraction lesions. Hence, in accordance with the criterion proposed by Zheng et al. [33], a force magnitude of F=225 N and F=405 N are applied in load cases ORV, OLV, BRV, and BLV, c.f. Table 2 and Figure 3.

Thus, $F_{N}=225$ N is imposed on the top edge of the tooth with an angle of θ=11° apart from the vertical line. On the other hand, $F_{a}$ is a horizontal force with a magnitude of $F_{a}=90$ N at each side, which means in BRV and BLV load cases, the magnitude of the horizontal load is F=180 N in total, c.f. Figure 3.

Regarding the computational model, it has been prepared in a FEM-based code software, FEMAP^©^-2017. It must be mentioned that the nodes/elements coordinates respecting their connectivity have been imported as input into the developed numerical algorithms implemented in MATLAB^©^-R2017a. The numerical algorithms were fully programmed by the authors based on a FEM code using the FE mesh background to establish the meshless nodal discretisation. Therefore, boundary conditions and material properties were fully defined and applied to the model, then the nodal connectivity, nodal integration, and the shape functions were implemented in accordance with each method’s requirements.

Concerning the FEM model, the problem domain has been discretised through the standard 2D constant strain triangle elements of type S3. Hence, the same discretisation was considered maintaining nodal points to be analysed by both meshless methods. Figure 2a shows the numerical model with the FE mesh representation. The model includes 4968 elements and 2467 nodes. Since the model possesses an irregular geometry, S3 elements were selected to generate the mesh on the model. Therefore, it allows for the generation of meshes capable of accommodating highly irregular boundaries. This type of mesh proved to be a proper mesh for the FEAs as demonstrated in the previously published paper [34]. It must be noted that each influence domain in meshless method analyses possesses 20 nodes to acquire reliable results with reasonable computational costs, as recommended by Farahani et al. [35].

Likewise, these elements are appropriate for the RPIM, since they allow construction of the background integration mesh. Regarding the NNRPIM, since the formulation only requires the nodal mesh of the model, the element mesh is discarded. In addition, the dimensional approach of the numerical analysis is a 2D plane strain; see Figure 1.

## 5. Analysis and Results

The numerical analyses have been performed on the model using RPIM and NNRPIM meshless methods and the FEM (for validation purpose). The equivalent von Mises effective stress $\left(\sigma_{v}\right)$ results were obtained for a normal tooth (denoted as integral material) and restored teeth associated with three different composite materials.

**Figure 4:**
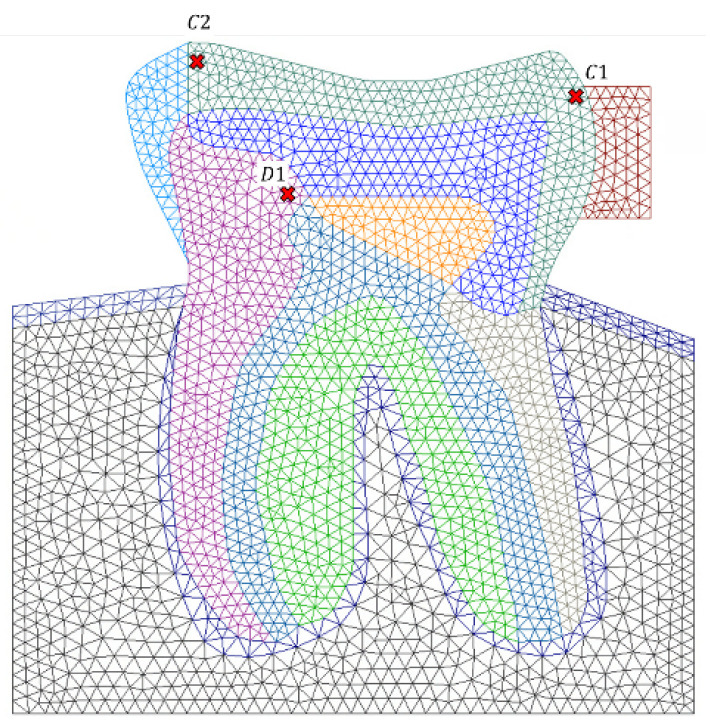
Interest points defined on the model to monitor the local stress.

In an elasto-static analysis, finding hot spots in terms of the magnitude of the stress is a crucial stage of the study. For instance, Mehri Sofiani et al. [36] identified the most critical regions in a FE model by accounting for the stresses higher than the 98% of the highest principal stress in a region of interest. In this work, to assess the local stress, referring to Figure 4, some interesting spots were selected in the model in which three points {C1, C2, D1} were assigned at. Two critical cases were considered here; ORV and BRV, therefore, the local $\sigma_{v}$ has been extracted on the interest points acquired from all methods, as accordingly presented in Table 3 and Table 4.

It must be mentioned that D1 is in a region permanently associated with dentine material while C1 and C2 can be filled by enamel or any composite resin. Integral case refers to the one in which the corresponding section, referring to Figure 2a, were filled with the natural material as follows: section 1: enamel; section 2: dentine; and section 3: pulp. On the other hand, case Z100^TM^ means that the corresponding sections were restored with the Z100™.

Referring to the reported results for ORV and BRV cases, generally, there is a reasonable agreement amongst the results achieved for composite materials. It must be remarked that there is a difference between the meshless and FEM solutions. This difference could be explained by the curvature in the corresponding region, which is related to the complexity existing locally in the geometry. Therefore, FEM would lose its accuracy in such locations and meshless methods seem more reliable due to the nodal distribution.

The local stress accounted for a higher value in the BRV case compared to the ORV since the force magnitude is greater in the BRV case study and the force was transversally imposed in addition to its imposition with an angle to the tooth’s top surface, denoting a mixed mode condition. Regardless the material assignment and employed numerical methods and considering $\sigma_{v}^{max}=70$ MPa, it is feasible to provide a deep discussion on the results acquired from local stress on both case studies. Concerning the ORV case, in which a magnitude of $F_{N}=225$ N load is applied with an angle from left to right direction, the fictious material significantly resists against the subjected load, therefore, point $D_{1}$ tolerated a relatively high tensile stress concentration. Its induced stress is around 30% of the maximum stress accumulation. However, C1 underwent a compressive stress state upon loading due to its position adjacent to the fictious material with a stress level of approximately 10% of maximum value. Regarding C2, it is a bit far away from the fictious material location, tolerating a relatively small stress of less than 3% of the maximum stress.

On the other hand, in the BRV case study, the scenario is different. A horizontal load of $F_{a}=90$ N is present in addition to $F_{N}=225$ N, which is dominant in the ORV case. Therefore, point C1 encountered the maximum compressive stress since it is located in the loading direction and next to the fictious material, with a stress level of more than 50% of $\sigma_{v}^{max}$. Regarding points C2 and D1, the stress state is moderate, being more relaxed compared to C1, roughly around 30% of the maximum stress level.

If material assignment is taken into account, the integral material reflects a strong resistance against stress application due to the superior elasticity modulus of enamel and dentine material compared to the composites. With a closer look into the results, it is possible to assess each composite material in terms of stress analysis. For all interest points, a rank can be classified on both case studies, ORV and BRV, for the composite resins regarding von Mises effective stress as follows:

Z100^TM^ > Z250^TM^ > Herculite XRV Ultra^TM^

Practically, tensile, and compressive strengths serve as indicators of material strength under distinct force conditions, with stronger materials exhibiting higher values. Materials with high compressive strength offer prolonged resistance against heavy loads, especially in posterior restorations. Herculite XRV Ultra^TM^ reveals a weak response in the stress analysis due to its lowest mechanical properties amongst all studied composites; it is less stiff. The Z100^TM^ microhybrid composites displayed superior behaviour compared to others. This is because of their elevated filler loadings. Increasing filler content was observed to correlate with heightened hardness, compressive strength, and, thereby, stiffness; this topic has been fully addressed and confirmed by Curtis et al. [37]. It is noteworthy that the trend in filler size reduction from traditional to nano-hybrid materials, driven by the pursuit of aesthetic properties, has implications. The larger surface-area-to-volume ratio in nano-filled materials may lead to increased water uptake, potentially degrading the filler/matrix interface and affecting mechanical properties in comparison to microhybrid composites [37].

For the ORV load case, for C1, the integral material accounted for lower stress compared to all composites while this scenario was reversed for C2. This may happen due to the boundary conditions, since the mentioned points are located very close to the boundaries.

Generally, the results for the BRV load case indicate that the von Mises stress is increased for point C1 since it is distributed on the border between tooth and the composite material and due to the presence of the neighbour tooth reflecting the applied force. Therefore, conforming to the force application, C2 is under tension while C1 is under compression. Thus, C1 tolerates more stress compared to C2.

More observations from the BRV results in relation to D1 are associated with dentine material, since it reflects a mixed mode loading condition. In fact, the elastic modulus of enamel is about three times greater than that of dentine. Because the elastic modulus represents the ratio of the elastic stress to the elastic strain, it follows that the lower the strain for a given stress, the greater the value of the modulus. Dentine can sustain significant deformation under compressive loading before it fractures. On the other hand, enamel is a stiffer and more brittle material than dentine and unsupported enamel is more susceptible to fracture. Conversely dentine is more flexible and tougher. Nevertheless, the proportional limit, compressive strength, and elastic modulus of enamel are greater than the corresponding values for dentine, c.f. [29,38].

**Figure 5:**
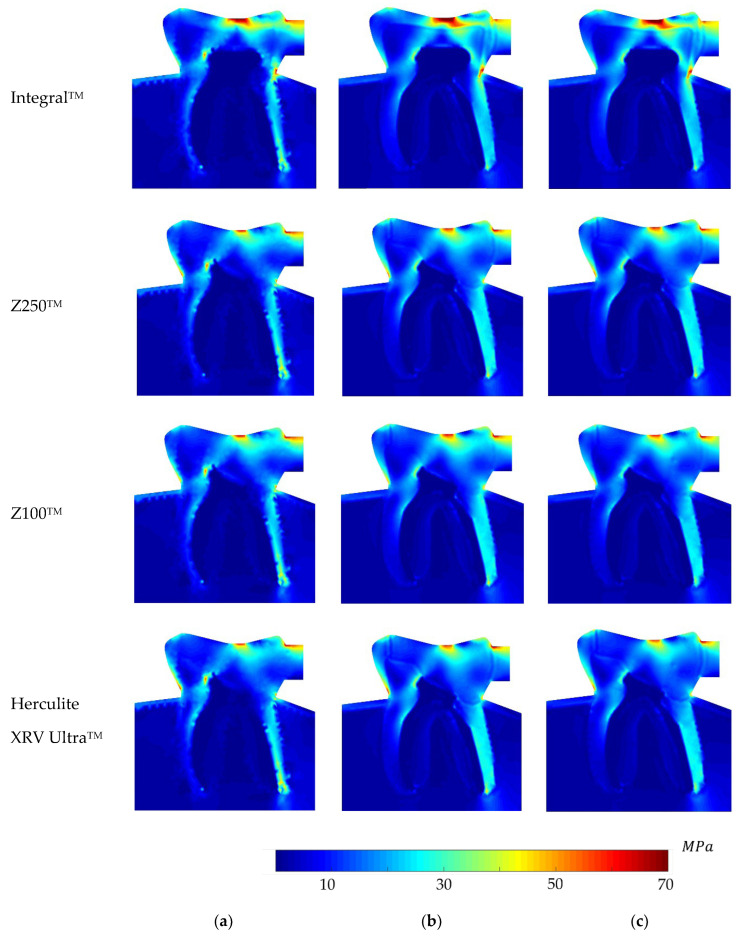
Global von Mises effective stress profile for the BRV load case for integral and different composite materials, (**a**) FEM, (**b**) RPIM, and (**c**) NNRPIM meshless methods.

**Figure 6:**
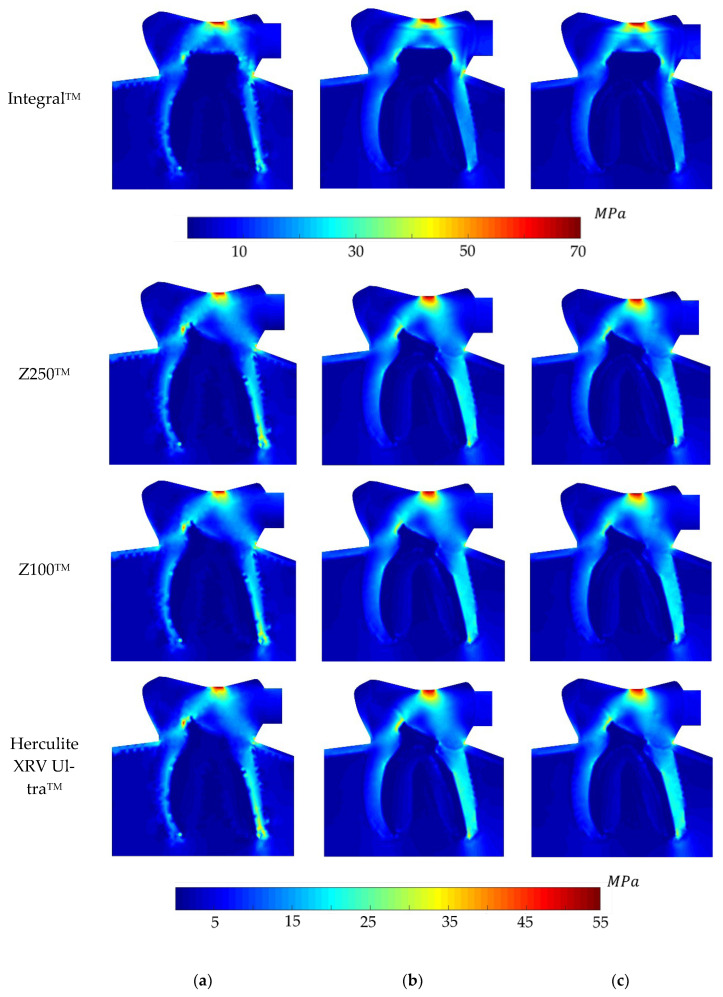
Global von Mises effective stress profile for the ORV load case for integral and different composite materials, (**a**) FEM, (**b**) RPIM, and (**c**) NNRPIM meshless methods.

**Figure 7:**
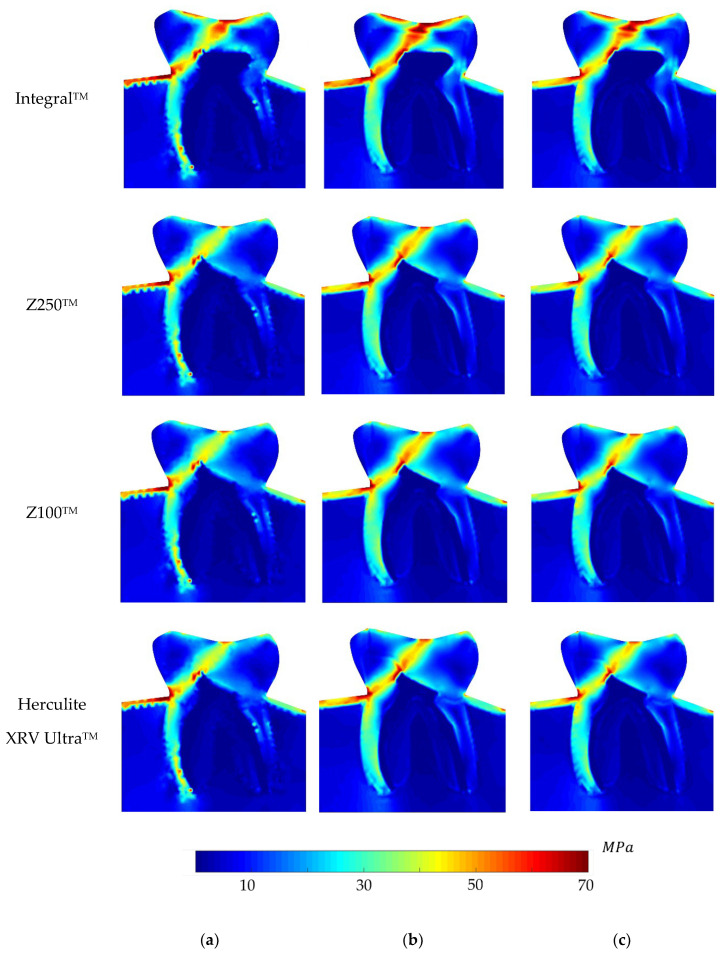
Global von Mises effective stress profile for the BLV load case for integral and different composite materials, (**a**) FEM, (**b**) RPIM, and (**c**) NNRPIM meshless methods.

**Figure 8:**
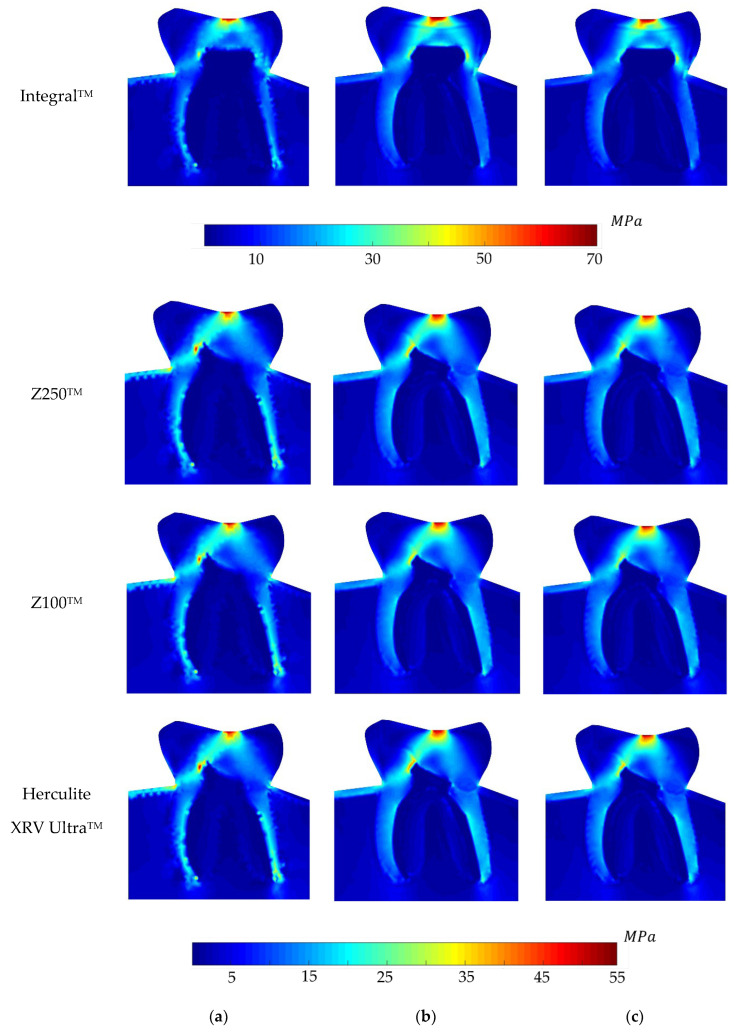
Global von Mises effective stress profile for the OLV load case for integral and different composite materials, (**a**) FEM, (**b**) RPIM, and (**c**) NNRPIM meshless methods.

Regarding the equivalent von Mises effective stress contour on the whole problem domain, Figure 5, Figure 6, Figure 7 and Figure 8 demonstrate the global stress profiles for BRV, ORV, BLV, and OLV case studies, respectively, obtained from all numerical analyses for integral (natural tooth material) and three composite materials in the case of tooth restoration.

## 6. Discussion on the Results

Overall, there is an effective verification among the global stress profiles obtained from the FEM and meshless methods. However, it is noteworthy that both RPIM and NNRPIM meshless methods exhibit strikingly similar stress contours in terms of shape and smoothness. More precisely, according to the obtained contours, it can be inferred that the stress accounted for a high value mostly on the borders, close to the boundaries, either natural or essential ones.

The maximum stress for OLV, ORV, and BRV is in the zone under oblique load. On the other hand, in the BLV load case, the maximum stress distribution is mostly on the left side of the tooth, starting from the region under oblique load, before smoothly becoming curved and travelling to the cortical bone. The spot on the border between the cortical bone and the dentine of the tooth is standing the highest amount of stress.

The stress in the integral material accounted for a higher magnitude compared to the composites in both ORV and OLV case studies from all analyses. This phenomenon can happen due to the material homogeneity under the defined boundary conditions, since the whole tooth is structured by integral materials with different natural properties. Additionally, it can be concluded that the stress is intensive in the loading position since there is only a slanted force imposed on the tooth’s top surface. The high severity of stress is continuing through the loading direction into the material. It can be inferred that the boundary condition defined on the fictitious material (right side of the studied tooth) in the ORV case does not affect the stress distribution since the loading condition stayed the same.

Regarding the BRV case study, it can be implied that the transverse load leads to accumulating higher stress in the position where fictitious material exists considered as a fixed boundary condition. This phenomenon is more remarkable if integral material is considered. However, the loading position underwent a relatively higher stress value, and it became lower if the distance was further.

Stress analysis in the BLV case is relatively different from the other case studies. The tooth is completely free on lateral sides and a force with an angle is applied to the top surface in addition to two transverse loads imposed in the structure. Therefore, the stress was concentrated at the slanted loading position and its high intensity is following the load path through the material. Then, the tooth’s left side (which is in the loading direction) experienced a high-stress accumulation, especially on the interface to the cortical bone. It must be noted that this zone is susceptible to wedge-shaped defect formation as diagnosed in [39]. Likewise, the integral material tolerated more intensive stress accumulation compared to the composite materials. The regions with the highest stress concentration are the most susceptible ones to fracture, as they would already exceed the material ultimate strength.

In summary, the BLV case would be considered as the worst-case scenario compared to the other cases. The global stress distribution maps obtained with the meshless method clearly show the potential rupture lines.

## 7. Conclusions

A 2D elasto-static analysis of a human molar tooth under different loading conditions was studied by meshless methods compared to the FEM solutions. From the clinical point of view, it focuses on bruxism, which is considered one of the most common dental disorders to cause the fracture of the teeth. Natural tooth and restored tooth are considered as the studied model. The molar tooth is divided into different sections to assign heterogeneous material properties and three sections were filled by integral and three different composite materials Z100^TM^, Z250^TM^, and Herculite XRV Ultra^TM^. Owing to the obtained results, the following conclusions could be drawn:

- The non-restored tooth is characterised by enamel rather than composite restoration. Enamel exhibits the highest Young’s modulus compared to the composites. Consequently, an integral tooth demonstrates a greater ability to withstand forces compared to composite restorations. It means that the stiffness of enamel is higher than that of the restorative materials.
- The ranking on the local stress analysis can be rationalised based on the superior mechanical properties of Z100^TM^, including its elevated Young’s modulus, notably higher tensile and compressive strength compared to other composite materials. This implies that dental restoration using Z100^TM^ results in increased stiffness and rigidity in the treated tooth. It owns the highest percentage of fillers, 66%, which implies higher hardness.
- Herculite XRV Ultra^TM^ exhibited the weakest response in terms of elasto-static analysis. One potential reason could be attributed to its lower Young’s modulus and consequently, the lowest hardness, which may be influenced by the presence of barium glass filler in this composite. Conversely, the Zirconia Filler in Z100^TM^ and Filtek Z250^TM^ might account for the improved physical properties observed in these two composites.
- In the depicted stress profiles of the BLV load case, where there is no tooth on the left side of the model (no essential boundary condition), the stress distribution shows higher value on critical regions, mostly close to the boundaries. The absence of essential boundary conditions on the left side of the 2D model in the BLV and OLV load cases leads the applied force to produce a higher level of stress on the borders and critical spots. In this regard, the global stress distribution maps obtained with the meshless methods clearly show the potential rupture lines. This study shows the importance of the neighbour tooth to prevent a potential fracture.
- There is an acceptable verification between the FEM and meshless results. However, in some cases, in the points close to more complex curves, FEA yields a far different value from the ones calculated by meshless methods.
- Within the restrictions of the numerical methods, the computational simulations implemented in this work have the capacity to improve and refine the results until they are closer to clinical implications. Performing these simulations with denser meshes could potentially lead to better results. Furthermore, future composite materials for dental restorations perhaps will possess better mechanical properties, especially a higher Young’s modulus. Thus, mechanical improvement on composites will eventually lead them to withstand critical loads of bruxism. It is undeniable that deeper research on this topic would allow increases in the quality of patients’ life.

## Figures and Tables

**Table 2:** Force magnitude for each load case.

Load Case	Force Magnitude (N)
ORV	225
OLV	225
BRV	405
BLV	405

**Table 3:** Equivalent von Mises effective stress obtained at interest points by FEM and meshless methods, ORV case.

		$\sigma_{v}$ (MPa)			
Point	Method	Integral	Z100^TM^	Z250^TM^	Herculite XRV Ultra^TM^
C1	FEM	5.59	8.21	7.43	6.59
	RPIM	3.19	6.04	5.15	4.25
	NNRPIM	3.06	5.68	4.86	4.03
C2	FEM	3.00	1.57	1.55	1.53
	RPIM	2.93	1.24	1.17	1.11
	NNRPIM	2.75	1.15	1.08	1.03
D1	FEM	16.47	20.59	20.34	20.19
	RPIM	23.95	22.46	21.43	20.33
	NNRPIM	26.73	23.68	22.59	21.45

**Table 4:** Equivalent von Mises effective stress acquired at interest points by FEM and meshless methods, BRV case.

		$\sigma_{v}$ (MPa)			
Point	Method	Integral	Z100^TM^	Z250^TM^	Herculite XRV Ultra^TM^
C1	FEM	39.71	38.62	37.91	37.01
	RPIM	38.36	38.50	37.11	35.58
	NNRPIM	36.86	36.24	34.73	33.08
C2	FEM	25.35	22.18	21.38	20.50
	RPIM	29.96	22.51	20.83	19.13
	NNRPIM	27.03	19.99	18.34	16.64
D1	FEM	17.81	21.34	20.92	20.54
	RPIM	23.74	21.03	19.63	18.09
	NNRPIM	26.48	22.39	20.89	19.25

## Data Availability

The data presented in this study are available on request from the corresponding authors.

## Nomenclature

2D	Two-dimensional	RPIM	Radial point interpolation method
3D	Three-dimensional	RPI	Radial point interpolation
BisGMA	Bisphenol A-glycidyl methacrylate	TEGDMA	Triethylene glycol dimethacrylate
BRV	Bruxism right vertical	UDMA	Urethane dimethacrylate
BLV	Bruxism left vertical	E	Young’s modulus
CNS	Central nervous system	F	Applied force
FEA	Finite element analysis	ν	Poisson’s ratio
FEM	Finite element method	σ	Stress tensor
NNRPIM	Natural neighbour RPIM	$\sigma_{c}$	Compressive strength
OLV	Occlusal left vertical	$\sigma_{t}$	Tensile strength
ORV	Occlusal right vertical	$\sigma_{v}$	Equivalent von Mises effective stress
